# The Association of lncRNA and mRNA Changes in Adipose Tissue with Improved Insulin Resistance in Type 2 Obese Diabetes Mellitus Rats after Roux-en-Y Gastric Bypass

**DOI:** 10.1155/2022/8902916

**Published:** 2022-07-18

**Authors:** Li-Hai Zhang, Jiao Wang, Bai-Hong Tan, Yan-Bin Yin, Yu-Ming Kang

**Affiliations:** ^1^Department of Physiology and Pathophysiology, Xi'an Jiaotong University School of Basic Medical Sciences, Xi'an 710061, China; ^2^General Surgery, The First Affiliated Hospital of Jiamusi University, Jiamusi, Heilongjiang 154002, China

## Abstract

**Objective:**

Roux-en-Y gastric bypass (RYGB) has shown good effects in improving obesity and type II diabetes mellitus (T2DM), but the underlying mechanisms remain unclear. This study explored the changes of related lncRNAs, mRNAs, and signaling pathways in white adipose tissue of T2DM rats after RYGB based on RNA-Seq sequencing, with the aim to provide a theoretical basis for RYGB treatment.

**Methods:**

T2DM rat models were established by continuous feeding with a high-fat diet and injection of streptozotocin (STZ), after which they underwent RYGB or sham surgery. After the surgery, their body weight was measured weekly. Their fasting blood glucose (FBG) and fasting serum insulin (FSI) were also measured. A homeostasis model assessment of insulin resistance (HOMA-IR) was calculated at weeks 0, 8, and 12. Besides, white adipose tissue of T2DM rats was collected for RNA-Seq sequencing and validated by qRT-PCR. A series of bioinformatics analyses, such as differential expression genes (DEGs) screening, was performed. GO and KEGG functional enrichment analysis and protein-protein interaction (PPI) network construction were conducted based on the sequencing data.

**Results:**

RYGB surgery could significantly inhibit the weight growth rate and decrease the FBG, FSI, and HOMA-IR of T2DM rats. Bioinformatics analysis of RNA sequencing (RNA-Seq) results revealed that 87 DE- lncRNAs (49 upregulated and 38 downregulated) and 1,824 DEGs (896 upregulated and 928 downregulated) were present in between the RYGB group and Sham group. GO and KEGG analysis showed that the target genes of DEGs and differentially expressed lncRNAs (DE-lncRNAs) were mainly associated with amino acid metabolism, fatty acid metabolism, channel activity, and other processes. In addition, the PPI network diagram also displayed that genes such as Fasn, Grin3a, and Nog could be key genes playing a role after RYGB. qRT-PCR showed that the expression level of Grin3a in the RYGB group was significantly increased compared with the Sham group, while the expression of Fasn and Nog was significantly decreased, which was consistent with the sequencing results.

**Conclusion:**

Using RNA-Seq sequencing, this study revealed the changes of related lncRNAs, mRNAs, and signaling pathways in the white adipose tissue of T2DM rats after RYGB and identified Fasn, Grin3a, and Nog as potential key genes to function after RYGB.

## 1. Introduction

Epidemiological studies have reported that diabetes mellitus (DM) will become an epidemic accompanied by metabolic and endocrine disorders worldwide. Most DM patients are affected by type 2 diabetes mellitus (T2DM), with insulin resistance and secretion defects [1, 2]. The incidence of T2DM has significantly increased over the past 10 years and is estimated to affect 439 million people by 2030 [3] T2DM can increase the risk of various diseases such as cardiovascular complications and certain cancers, increasing the risk of mortality and morbidity, and leading to a worsening economic burden [4]. It is worth noting that the rapid increase in the incidence of obesity worldwide has also induced a parallel increase in T2DM cases. Obesity is characterized by excessive body fat accumulation and storage, with white adipose tissue (WAT) occupying 50% of body weight and playing an important secretory role. Studies have demonstrated that WAT plays a major role in the pathogenesis of obesity-related insulin resistance by secreting several inflammatory adipokines that regulate obesity-induced insulin resistance [5]. Currently, commonly applied lifestyle-oriented methods and pharmacological interventions are not satisfactory for treating T2DM [6].

Gastric bypass surgery was initially developed in the 1960s and was based on the weight loss observed among patients undergoing partial stomach removal for ulcers [7]. Over several decades, the gastric bypass was modified into its current form, the Roux-en-Y gastric bypass (RYGB). It is used in treating several diseases such as stomach ulcers [8], gastric cancers [9], and weight loss [10]. RYGB surgery has been found to be effective in promoting sustained weight loss, improving glycemic control, and reducing cardiometabolic risk factors. The efficacy of RYGB and sleeve gastrectomy was found to be superior compared with medical treatment for treating T2DM in prospective randomized clinical trial settings [11, 12], which led to the consensus of bariatric surgery in the treatment algorithm of T2DM. Nonetheless, there is some controversy in the application of RYGB due to its unclear mechanism of action in treating T2DM. Therefore, it is necessary to elucidate the mechanism of RYGB and provide theoretical support for the treatment of T2DM.

Long noncoding RNAs (lncRNAs) are RNA molecules with a length of more than 200 nucleotides but do not have protein translation ability [13, 14]. At present, approximately 27,919 human lncRNAs have been reported, of which 70% are functional. LncRNAs can interact with proteins, RNAs, and DNA molecules in cis- and transregulatory activities to regulate transcriptional processes and participate in various metabolic pathways in cells or the body, including the development of T2DM [15]. Arner et al. reported 51 differentially expressed lncRNAs (DE-lncRNAs) in WAT of insulin-resistant patients in obese women with systemic insulin resistance and revealed a correlation between these genes and cellular metabolism [16]. Gao et al. discovered that lncRNA ASMER not only promoted insulin resistance in the liver of obese rat models but also caused excessive fat accumulation by affecting PPARG and INSR [17]. Overall, these studies showed that aberrantly expressed lncRNAs and mRNAs in WAT might play vital roles in T2DM development.

Interestingly, Liang et al. compared lncRNA expression profiles in the duodenum of T2DM patients with or without RYGB and found that duodenal lncRNA was linked to glycemic control after RYGB in high-fat diet-induced diabetic mice [18]. Therefore, it can be speculated that RYGB may improve insulin resistance by affecting lncRNA and mRNA expression in patients with T2DM after RYGB. However, the effects of RYGB on lncRNA and mRNA expression in WAT of T2DM are still unclear. In this study, we tried to reveal lncRNA and mRNA changes in adipose tissue via RNA sequencing (RNA-Seq) and bioinformatics analysis to provide a theoretical basis for elucidating the mechanism of RYGB.

## 2. Materials and Methods

### 2.1. Establishment of Rat Model of Type 2 Diabetes Mellitus and Roux-en-Y Gastric Bypass Surgery

This study was approved by the Ethics Committee of Guangdong Experimental Animal Research Center and conducted following the approved guidelines. A total of 24 8-week-old male SD rats (weighing 180–220 g) were purchased from Beijing HFK Biosciences Co., Ltd. The rats were randomly divided into four groups after 1 week of adaptive feeding, and a rat model of T2DM was constructed and treated with RYGB according to the method in previous studies [19, 20]. Rats in the normal group (*n* = 8) were fed a standard diet (18% fat, 25% protein, and 57% carbohydrate) for 4 weeks, followed by an intraperitoneal injection of 30 mg/kg of sodium citrate buffer into each rat. Rats in the Sham group and RYGB group (*n* = 16) were fed a high-fat diet (40% fat, 13% protein, and 47% carbohydrate) for 4 weeks, followed by an intraperitoneal injection of 30 mg/kg streptozotocin (STZ). After another 4-week of feeding, the random blood glucose of the rats was measured to be higher than 16.7 mmol/L, suggesting the successful construction of the rat model of T2DM. Sixteen T2DM rats were randomly assigned to the Sham and RYGB groups, with 8 rats in each group. T2DM rats were fasted on the day before RYGB. They were then anesthetized with 1% pentobarbital sodium solution (5 ml/kg) for midline laparotomy under sterile conditions. A GIA stapler (ETS-Flex Ethicon Endo-Surgery 45 mm) was utilized to dissect their stomach to create a 20% gastric pouch. The small intestine was dissected to make a 15-cm biliopancreatic limb, a 10-cm digestive tract (Roux) limb, and a 33-cm common channel. Gastrojejunostomy and jejunojejunostomy were performed with interrupted 5-0 silk sutures, followed by abdominal closure with 3-0 silk and 5-0 propylene. Rats in the Sham group received similar preoperative or postoperative care as rats in the RYGB group. Diet and water were given about 24 hours after surgery, and the body weight of the rats was measured weekly. After 12 weeks, the rats in each group were euthanized, and WAT was taken from their groin.

### 2.2. Fasting Blood Glucose (FBG) and Serum Insulin (FSI) Tests

Fasting blood glucose (FBG) and serum insulin (FSI) were measured in the rats of each group at weeks 0, 8, and 12. The rats were fasted for one day on the day before the test, and FBG was measured in the blood of the rats with tail bleeding by Contour TS glucometer (Bayer, Germany). Blood samples were collected from the abdominal aorta of rats in each group, and after standing for 20 min, the samples were centrifuged at 4 °C, 2000 rpm for 10 min. Subsequently, the upper serum was collected, and the insulin content in the serum of rats in each group was detected using the corresponding insulin ELISA assay kit (Nanjing Jiancheng Bioengineering Institute, China). The homeostasis model assessment of insulin resistance (HOMA-IR) is calculated using the following formula: [FBG (mmol/l) × FINS (mU/l)]/22.5.

### 2.3. RNA Extraction, Illumina Sequencing, and Data Quality Control

Total RNA was extracted from 100 mg of WAT from each group of T2DM rats according to the RNA extraction kit instructions. The quality and quantity of extracted RNA were determined by a nanodrop spectrophotometer and an Agilent 2100 Bioanalyzer. RNA libraries were prepared from 250 ng of total RNA adopting the Illumina Exome Capture Kit. RNA-Seq was performed following the standard protocol of the DFCI Molecular Biology Core Facility (Illumina NextSeq 500).

### 2.4. Construction of lncRNA and mRNA Sequencing Libraries

After the total RNA extracted from the samples passed quality inspection, ribosomal RNA (rRNA) was removed using an epicenter Ribo-Zero kit (Illumina, USA), and the remaining part of rRNA depleted RNA (polyA + and polyA-) was purified and recovered. The purified and recovered RNA was randomly interrupted into short fragments by fragmentation buffer. The first strand of cDNA was synthesized with six-base random hexamers utilizing the interrupted short fragments as templates. Later, the buffer, dNTPs, RNaseH, and DNA polymerase I were adopted to synthesize the second strand of cDNA. Next, AMPure XP beads were applied to purify the double-stranded products. T4 DNA polymerase and Klenow DNA polymerase activities were employed to repair the viscous ends of the DNA to flat ends. At the 3′-ends, base A and adapters were added. AMPureXP beads were utilized for fragment selection, followed by degradation of the second strand of cDNA containing U with an USER enzyme. Lastly, PCR amplification was conducted to obtain the final sequencing library, and the library was sequenced using Illumina Hiseq4000 after passing the quality inspection. The sequencing read length was 2∗150 bp (PE150) at both ends.

### 2.5. Screening and Visualization of DE-lncRNAs and Differential Expression Genes (DEGs)

FPKM (fragments per kilobase of exon model per million mapped reads) was applied to measure the abundance values of gene expression of lncRNAs and mRNAs and to count the expression abundance of known genes in different samples. FPKM was equivalent to the expression of genes in different samples. Fold change ≥ 2 and *P* < 0.05 were the basic conditions for screening DE-lncRNAs and differential expression genes (DEGs). In addition, the R package DESeq2 was used to generate volcano plots of DEGs and DE-lncRNAs screening. VIPER (visualization pipeline for RNA-Seq analysis) was used to create a correlation heat map of intersample and display hierarchical clustering of Spearman rank correlations among the samples.

### 2.6. Gene Function Enrichment Analysis

The DAVID online database (https://david.ncifcrf.gov/) was utilized to analyze the functions of DEGs and enriched signaling pathways according to the cellular component, biological process, molecular function, and KEGG classification [21].

### 2.7. Protein-Protein Interaction Network Analysis (PPI)

Intergenic interactions were predicted by the STRING online website (https://string-db.org/), protein-protein interaction (PPI) networks were constructed based on gene overlap, and the data were imported into Cytoscape for visualization [22].

### 2.8. qRT-PCR

WAT of rats was collected and homogenized. Total RNA was extracted via the TRizol method, followed by detecting RNA concentration and purity by NanoDrop. Later, cDNA was prepared according to a random-primer reverse transcription kit (Thermo, USA). The expression level of Fras1, Grin3a, Osmr, Fasn, and Nog gene was detected referring to the instructions of the SYBR GREEN kit (TaKaRa, Japan); GAPDH served as an internal reference control, and six replicates were set up in the experiment. The 2 ^−*ΔΔ*Ct^ method was used to calculate the relative expression of the target gene based on experimental data obtained by qRT-PCR. The primer sequences used in this study are shown in Table 1.

### 2.9. Statistical Analysis

The test data were expressed as mean ± standard deviation (SD), and the SPSS 17.0 software was utilized for statistical analysis. One-way analysis of variance was adopted for comparisons among multiple groups, with *P* < 0.05 set as the threshold for statistical significance.

## 3. Results

### 3.1. Roux-en-Y Gastric Bypass Reduces Body Weight, Fasting Blood Glucose and Fasting Serum Insulin Level in T2DM Rats

**Figure 1:**
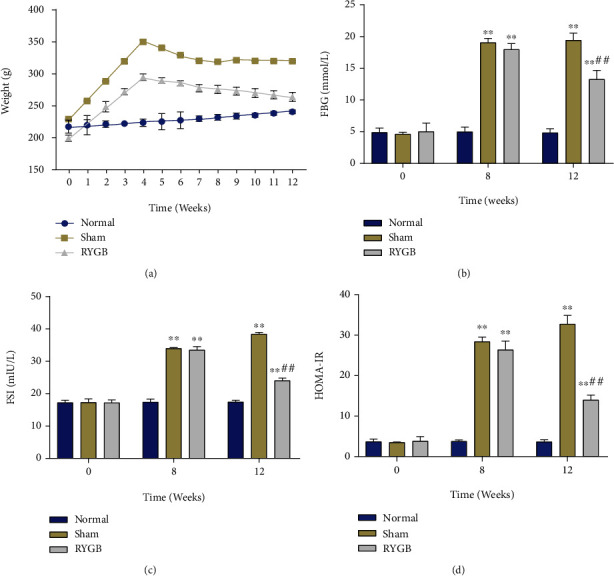
Roux-en-Y gastric bypass (RYGB) was associated with a reduction in body weight, fasting blood glucose (FBG) and fasting serum insulin (FSI) in type II diabetes mellitus (T2DM) rats. (a) Body weight of rats in each group measured weekly from 0 to 12 weeks. (b) Fasting blood glucose (FBG) level of rats in each group measured at weeks 0, 8, and 12. (c) Fasting serum insulin level (FSI) level of rats in each group measured at weeks 0, 8, and 12. (d) Homeostasis model assessment of insulin resistance (HOMA-IR) of rats in each group calculated by FBG and FSI, ^∗∗^*p* < 0.01, vs Normal; ##*P* < 0.01, vs sham.

To observe the effect of RYGB surgery on body weight, FBG, and FSI in T2DM rats, the T2DM rat model was first constructed by continuously feeding the rats with a high-fat diet and injection of STZ. Then, sham and RYGB surgeries were performed in the T2DM rats. By measuring the body weight of the rats in each group, we observed no significant difference in the baseline weight of rats in each group before surgery. The body weight of rats in the Normal group maintained a stable level from 0 to 12 weeks, while the body weight of T2DM rats in the Sham group exhibited a marked increase compared with the Normal group from 1 to 4 weeks. Also, the body weight of rats in the Sham group slowly decreased from 5 to 7 weeks and then remained stable, while that of rats in the RYGB group displayed a noticeable decrease from 8 to 12 weeks (Figure 1(a)). Additionally, FBG and FSI in rats were measured at weeks 0, 8, and 12, and the results indicated no significant differences in FBG, FSI, and HOMA-IR among the groups at week 0. Furthermore, FBG, FSI, and HOMA-IR in the Normal group maintained a stable low level at weeks 0, 8, and 12, while those in the Sham and RYGB groups were significantly higher than in the Normal group at weeks 8 and 12 (*P* < 0.01). Further, compared with the Sham group, the RYGB group exhibited a slight decrease in FBG, FSI, and HOMA-IR, while the decrease was significant at week 12 (Figures 1(b)–1(d)). These results suggested that RYGB could significantly reduce the body weight and FBG and FSI levels in T2DM rats.

### 3.2. Screening of DEGs and DE-lncRNAs in Adipose Tissue of T2DM Rats in the RYGB Group

**Figure 2:**
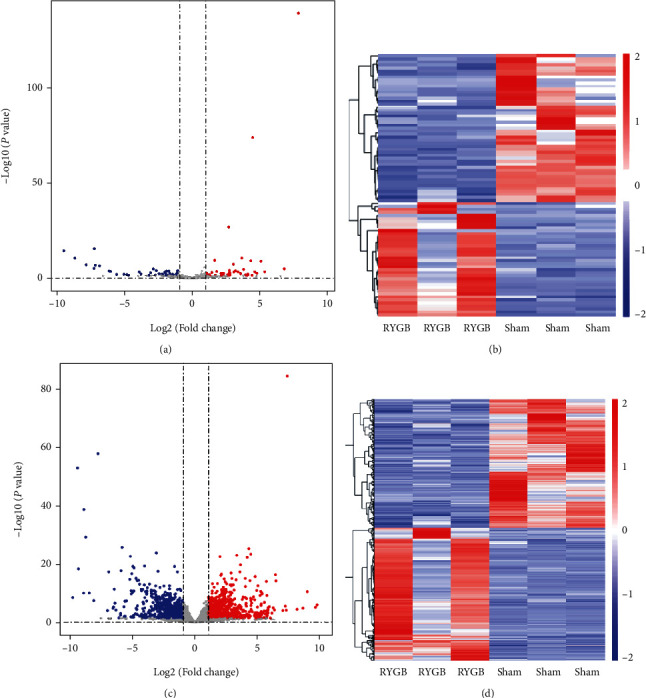
Screening of DEGs and DE-lncRNAs in adipose tissue of T2DM rats after RYGB. (a), (c) Volcano plot showing DE-lncRNAs (a) and DEGs (c) in adipose tissue of T2DM rats in the RYGB and Sham groups. (b), (d) Heat map showing DE-lncRNAs (b) and DEGs (d) in adipose tissue of T2DM rats in the RYGB and Sham groups. Blue represents downregulation; red represents upregulation.

To explore the mechanism of RYGB in reducing body weight, FBG and FSI in T2DM rats, we measured the genes and lncRNAs in adipose tissue of T2DM rats in the RYGB and Sham groups by RNA-Seq. Then, DEGs and DE-lncRNAs among the measured genes and lncRNAs were further screened. The results showed 87 DE-LncRNAs in the T2DM rats between the RYGB and Sham groups, including 49 upregulated lncRNAs and 38 downregulated lncRNAs (Figure 2(a)). According to the DE-lncRNAs heat map, the intragroup repeats were relatively homogeneous, and there were obvious separated clustering characteristics between the RYGB and Sham groups (Figure 2(b)). Similarly, 1,824 DEGs were also screened, including 896 upregulated and 928 downregulated genes (Figure 2(c)). Based on the heat map, relatively homogeneous expression can be observed in the RYGB and Sham groups, accompanied by obvious tissue clustering characteristics (Figure 2(d)). From the above, RYGB was associated with distinct lncRNA and mRNA expression in adipose tissue of T2DM rats compared with the Sham group and showed an independent trend.

### 3.3. GO and KEGG Functional Enrichment Analysis of DEGs

**Figure 3:**
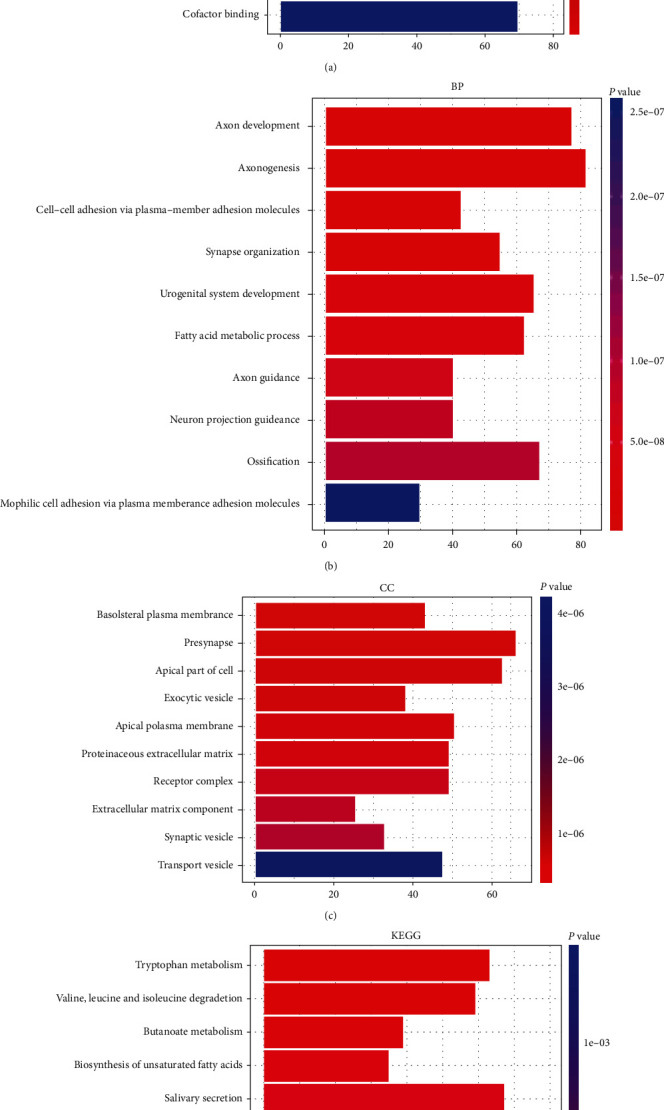
GO and KEGG functional enrichment analysis of DEGs. (a–c) GO analysis of related molecular functions (MF, a), biological processes (BP, b), as well as cellular components (CC, c) of DEGs in adipose tissue of T2DM rats in the RYGB group. (d) KEGG pathway analysis of enriched pathways of DEGs in adipose tissue of T2DM rats in the RYGB group.

To further investigate the functions and related pathways of DEGs in the RYGB group, we performed GO and KEGG functional enrichment analysis of DEGs in the RYGB group. In GO analysis, the results showed that DEGs in the adipose tissue of rats from the RYGB group were mainly associated with molecular functions such as various channel activities, transporter activities, compounds, proteins, and factor binding. Besides, DEGs were related to biological processes such as the occurrence and development of axons, the cell-cell adhesion and homophilic cell adhesion guiding and passing plasma membrane adhesion molecules, synaptic organization, and fatty acid metabolic processes. Moreover, DEGs were also correlated with cellular components such as vesicles, extracellular matrix, and receptor complexes (Figures 3(a)–3(c)). In addition, in KEGG, DEGs were principally enriched in various signaling pathways such as amino acid metabolism, fatty acid metabolism, gastric acid secretion, ECM-receptor interaction, and salivary secretion (Figure 3(d)).

### 3.4. Protein-Protein Interaction (PPI) Network Construction of DEGs

**Figure 4:**
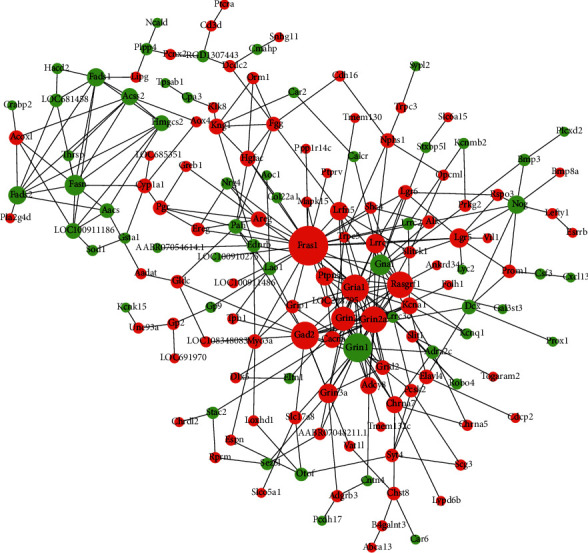
Protein-protein interaction (PPI) network construction of DEGs. Red represents increased expression, and green represents decreased expression.

To further screen the key DEGs, we predicted the interaction relationship among DEGs and mapped the PPI network through the STRING online database. The PPI network diagram showed that Fras1, Fasn, Grin3a, Nog, and Osmr were significantly associated with other differential genes, suggesting that they may play a relatively important role in the PPI network (Figure 4).

### 3.5. Interaction Network Analysis of DEGs and DE-lncRNAs

**Figure 5:**
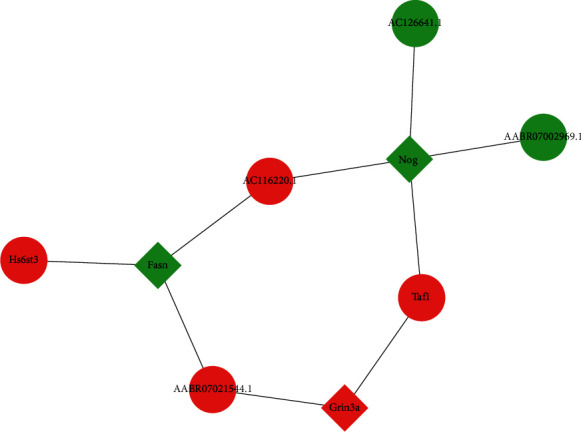
Network construction of key DEGs and DE-lncRNAs. Red represents increased expression, and green represents decreased expression.

Next, the targeting relationship between DE-LncRNAs and DEGs (Fras1, Fasn, Grin3a, Nog, and Osmr) was analyzed, and from the network graph, a direct targeting relationship of Fasn, Grin3a, and Nog with DE-LncRNAs could be observed (Figure 5).

### 3.6. qRT-PCR for Validating the Expression of DEGs in T2DM Rats

**Figure 6:**
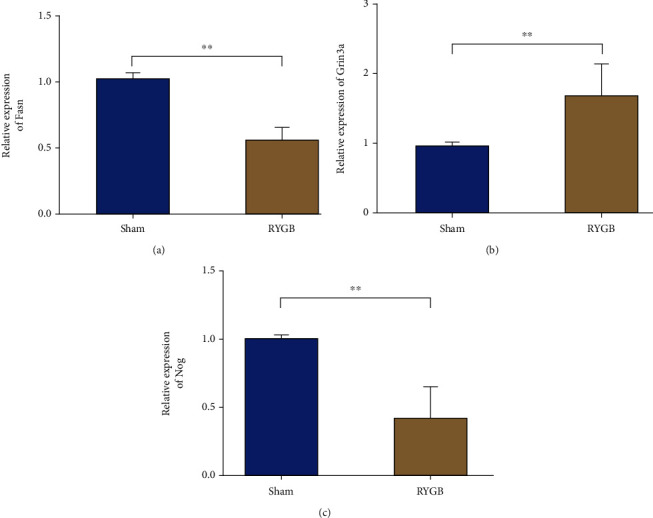
qRT-PCR for validating the expression of DEGs in T2DM rats in the Sham and RYGB groups. (a–c) qRT-PCR was used to verify the expression of Fasn (a), Grin3a (b), and Nog (c) in T2DM rats in the Sham and RYGB groups, ^∗∗^*p* < 0.01.

To validate the results of our high-throughput sequencing, qRT-PCR was adopted to verify the expression of the three DEGs—Grin3a, Fasn, and Nog—in the WAT of rats in the Sham and RYGB groups (Figures 6(a)–6(c)). The expression of Grin3a in WAT of the RYGB group was notably higher than in the Sham group (*P* < 0.01), while the expression of Fasn and Nog was markedly lower. The above results were consistent with the sequencing results, indicating the reliability of sequencing results.

## 4. Discussion

Several clinical studies have demonstrated that RYGB could greatly improve body weight and insulin resistance in patients with T2DM. Additionally, a 5-year randomized control trial by Courcoulas et al. showed that patients undergoing RYGB displayed the greatest mean decrease in 5-year body weight percentage, and insulin resistance was also improved [23]. The clinical trial by Cummings et al. also claimed that patients who underwent RYGB had a weight loss of 25.8 ± 14.5% after 1 year, a DM remission rate of 60.0%, and no life-threatening complications [10]. In this study, our results showed that RYGB could remarkably improve weight gain and insulin resistance in T2DM rats. All in all, RYGB surgery was shown to be a promising treatment for T2DM, but its mechanism remained underinvestigated.

With the popularization of high-throughput sequencing technology, more and more studies have demonstrated the key role of lncRNAs in the development of various diseases, including T2DM and obesity [24]. For example, Yang et al. reported that changes in the nutritional status of mice could lead to changes in the expression of lncRNAs in metabolism-related tissues (i.e., liver, WAT, and skeletal muscle) [25]. Besides, because some lncRNAs are present in circulating biofluid, some studies hold that these lncRNAs have the potential to act as biomarkers for T2DM. For instance, Carter et al. discovered that the serum level of lncRNA GAS5 was low in patients with T2DM, and a 12-fold higher risk of T2DM may occur in those with lncRNA GAS5 level that was less than 10 ng/*μ*L [26]. Additionally, related studies confirmed that lncRNAs in the peripheral circulation were mainly derived from WAT [27]. Consequently, we speculated that lncRNAs produced by WAT had a very powerful function.

Based on the above studies, we explored the molecular mechanism of RYGB associated with T2DM improvement. First, WAT was collected from rats that underwent RYGB and Sham surgery, and RNA-Seq was performed on the retrieved tissues. A total of 1,824 DEGs (896 upregulated and 928 downregulated) were identified by comparing the gene expression of WAT in the RYGB and Sham groups. The results showed that the expression of Fasn was notably downregulated in the RYGB group. It has been reported that despite causing an increase in the WAT synthesis of the body, the upregulation of Fasn expression is conducive to the recovery of insulin resistance [28]. Therefore, it can be speculated that RYGB can cause a reduction in the overall body weight by inhibiting the expression of Fasn. Currently, no study has investigated the role of the other two DEGs—Nog and Grin3a—in DM, so further experimental exploration is needed. Furthermore, GO analysis and KEGG pathway analysis showed that the differentially expressed mRNAs screened in this study mainly affected the processes like metabolism of amino acid, fatty acid, and pyruvate. Apart from the above, some studies have found that pyruvate metabolism was a major signaling pathway affecting T2DM formation [29], while other processes need further exploration.

Similarly, compared with the Sham group, the RYGB group had 87 DE-LncRNAs (49 upregulated and 38 downregulated) in WAT, while the function of most of the lncRNAs remained unknown. We also mapped the network of key DEGs and DE-lncRNAs, but most of the interaction relationships need further validation, and their functions remain to be clarified. Besides, we only explored DEGs, DE-lncRNAs, gene-related functions, and enriched signaling pathways. There could be many other potential mechanisms that have not been discovered yet and need further experimental validations.

## 5. Conclusions

In summary, RYGB surgery was found to be an effective treatment for T2DM. By RNA-Seq sequencing, 1,824 DEGs (896 upregulated and 928 downregulated) and 87 DE-lncRNAs (49 upregulated and 38 downregulated) were screened from the WAT of T2DM rats in the RYGB group. Additionally, GO and KEGG analyses revealed that the key DEGs were mainly associated with processes such as amino acid metabolism, fatty acid metabolism, and signaling activity. PPI network graph indicated that the Fasn, Grin3a, and Nog genes might be key genes affecting treatment efficacy after RYGB surgery.

## Figures and Tables

**Table 1:** Primers for quantification.

Gene	Sequences (5′ to 3′)
Grin3a	F: CAGCCAGAACTTGCTCTCCTTC
	R: GATGGAACCACTGAGACCTCTG
Fasn	F: TTCTACGGCTCCACGCTCTTCC
	R: GAAGAGTCTTCGTCAGCCAGGA
Nog	F: GATGGTCCTCAATGACTGGCAG
	R: GGACAACTTCCAAAGATGAGGAG
GAPDH	F: GTCTCCTCTGACTTCAACAGCG
	R: ACCACCCTGTTGCTGTAGCCAA

## Data Availability

The data used to support the findings of this study are available from the corresponding author upon request.
